# LEVERAGING A BEHEMOTH: TRANSLATING VA FAMILY CAREGIVER RESEARCH TO POLICY AND PRACTICE

**DOI:** 10.1093/geroni/igad104.0825

**Published:** 2023-12-21

**Authors:** Ranak Trivedi

**Affiliations:** Stanford University, Stanford, California, United States

## Abstract

The VA has been a pioneer in research, practice and policy that supports the needs of caregivers. The VA HSR&D funded Elizabeth Dole Center of Excellence in Veteran and Caregiver Research, of which Dr. Trivedi is a site PI and part of the core leadership, is a testament to this investment in research, as are several scientific projects. The VA also provides support for caregivers by providing an array of services (e.g., hotline; caregiver training programs; stipends) and has deployed 2000+ staff nationally to connect caregivers to VA and non-VA services. The VA has also invested in rigorous evaluations of existing practice and policy with the eye on refining and improving. Dr. Trivedi will provide an overview of her work within the VA that focuses on developing behavioral intervention to target the collective stress coping needs of Veteran-caregiver dyads. With the lens of implementation science, she will share her work identifying barriers and facilitators of accessing home and community-based services, and describe how these insights are being used to inform VA practice and policy.

